# Including personal boundaries scale: development and psychometric properties of a measurement for nurses' self-efficacy toward intimate and sexual behavior in dementia care

**DOI:** 10.3389/frdem.2024.1304438

**Published:** 2024-02-23

**Authors:** Karien Waterschoot, Tineke S. M. Roelofs, Arno van Dam, Katrien G. Luijkx

**Affiliations:** ^1^Tranzo, Scientific Center for Care and Welfare, Tilburg School of Social and Behavioral Sciences, Tilburg University, Tilburg, Netherlands; ^2^Psychogeriatric Psychology, Archipel Zorggroep Care Institute, Eindhoven, Netherlands; ^3^Research and Innovation, GGZ WNB Mental Health Institute, Halsteren, Netherlands

**Keywords:** scale, nurses, personal boundaries, dementia, intimacy, sexuality, nursing home

## Abstract

**Background:**

Person-centered nursing home care recognizes the intimate and sexual needs of residents with dementia but lacks guidance for nurses to address them while effectively respecting their personal boundaries. The Including Personal Boundaries (IPB) scale was developed to complement clinical and scientific efforts to support both nurse and resident wellbeing.

**Methods:**

Through a co-creative process, theoretical principles, day-to-day experiences, and expert knowledge were integrated into an initial nineteen-item version of the IPB scale. The pilot sample comprised 297 Dutch (vocational) nurses in dementia nursing care.

**Results:**

After Principal Component Analysis, nine items with strong factor loadings (>0.6) were retained. Internal reliability measures supported the item selection, such as high internal consistency (α = 0.866) and adequate corrected item-total correlations (0.532–0.781).

**Conclusion:**

The presented IPB scale, a nine-item scale, is a short, robust measure to assess nurses' self-efficacy in their capabilities to include personal boundaries (physical and emotional) when confronted with the intimate and sexual behaviors of residents with dementia. Further validation is recommended. The IPB scale could provide valuable insights for research, clinical practice, and education.

## 1 Introduction

Dementia affects nursing home residents' ability to empathize and communicate with their nurses, yet they highly depend on them in all aspects of their lives (Alsawy et al., 2020). Worldwide, nursing homes (NHs) tend to embrace a Person-Centered care (PCC) approach in their care for residents with dementia (Kim and Park, 2017; Fazio et al., 2018b). Key to the PCC philosophy is the recognition and affirmation of the “whole” person with dementia (Fazio et al., 2018a). In practice, PCC is a socio-psychological treatment approach built around individual needs and contingent upon close care relations (Koren, 2010). These relations should enable nurses to (1) interpret the unmet needs of residents and (2) tailor care to meet these needs (Fazio et al., 2018a).

PCC-labeled interventions have been improving residents' quality of life, contributing to an ongoing increase in job satisfaction among nurses (Kim and Park, 2017; Rajamohan et al., 2019). However, the ambiguity of the PCC philosophy poses challenges in both scientific and clinical operations (Wilberforce et al., 2016; Byrne et al., 2020). This ambiguity encompasses the absence of clear definitions concerning boundaries, specifically, the (professional) limits related to (1) need fulfillment, and (2) relational closeness. Professional intimacy (PI) is closely linked to close care relations in PCC. Antonytheva et al. (2021) defines PI as the therapeutic relationship between a nurse and a resident that fosters closeness, self-disclosure, reciprocity, and trust. For many nurses, shaping a caring relationship through PI that remains comfortable for themselves represents an ongoing challenge as they attempt to walk the fine line between meeting a resident's (care) needs and becoming too personally involved (Peternelj-Taylor and Yonge, 2003). As nurses become overly personally involved, they may instinctively respond to residents, inadvertently allowing both parties to compromise the relationship in the long run. For instance, nurses may engage in excessive self-disclosure, impose personal norms, or initiate unwarranted physical touch. Conversely, nurses may also find themselves struggling to halt or redirect these actions from residents (Peternelj-Taylor and Yonge, 2003; Baca, 2011). For residents with dementia, this may be confusing as the nurses' behavior can be interpreted as more familiar than the nurses intended.

This challenge is especially evident when it comes to the intimacy and sexuality of residents with dementia. Though their intimate and sexual needs and expressions are diverse, most feel limited in their abilities to express themselves sexually within the NH (Roelofs et al., 2021). Nurses, encounter a wide range of verbal and physical intimate and sexual behavior, such as hugs, kisses, masturbation, and even harassment (Makimoto et al., 2015). Naturally, nurses, being human, shape their personal interpretation of behavior, and establish personal boundaries through their values, beliefs and past experiences. Moreover, the interpretation of intimate and sexual behavior, and the decision whether this behavior exceeds the personal boundary, also appears to be influenced by the resident expressing the behavior. Unwanted expressions from residents thus generate varying degrees of emotional distress and insecurity among nurses (Zwijsen et al., 2014; Waterschoot et al., 2021). In this study, we define these feelings, and sometimes related bodily experiences, as nurses' personal boundaries. To integrate them into care, nurses must be able to act upon these evoked feelings within the context of a caring relationship, which is an integral aspect of their job and a protective mechanism against distress and possibly even burnout (Kokkonen et al., 2014; Yao et al., 2018; Rapp et al., 2021). However, PCC theory lacks clear guidelines for nurses, especially in navigating situations involving intimate and sexual behavior (Vandrevala et al., 2017).

Research suggests that, while nurses typically demonstrate an accepting attitude toward residents' sexuality in general, they may struggle to respond in a calm and respectful manner when sexual expressions are directed toward them (Pinho and Pereira, 2019; Roelofs et al., 2019; Villar et al., 2020). When nurses, on the other hand, do feel capable of including personal boundaries, they enact coping strategies that regulate their upcoming emotions and, subsequently manage the interaction between them and the resident (Biggs et al., 2017). When nurses perceive that their boundaries have been violated, it can lead to negative effects, including stress, diminished mental health, or resident aversion (Nielsen et al., 2010; Lu et al., 2020; Waterschoot et al., 2021). This can impact the care relationship, as nurses may either become too close or too distant to tend to the wellbeing of both parties (Mcguire et al., 2016).

Being aware of and acting upon personal boundaries appears to be the helping pathway for both residents and nurses, as it fosters a stable care relationship where the interests of both parties are harmonized. A scale measuring nurses' capabilities to include personal boundaries regarding the intimate and sexual behavior of residents could support the guidance, assistance, and education of NH nurses. Furthermore, such a scale might enable research to shed a more nuanced light on the complex task nurses face in delivering PCC across all aspects of a resident's life, including intimacy and sexuality. To the best of our knowledge, there are currently no scales available that measure caregivers' (perceived) ability to include personal boundaries during care. Therefore, this study aimed to develop, test, and validate a self-efficacy scale that accurately measures nurses' confidence levels in their capabilities to include personal boundaries (physical and emotional) when confronted with the intimate and sexual behavior of nursing home residents with dementia.

## 2 Methods

**Figure 1:**
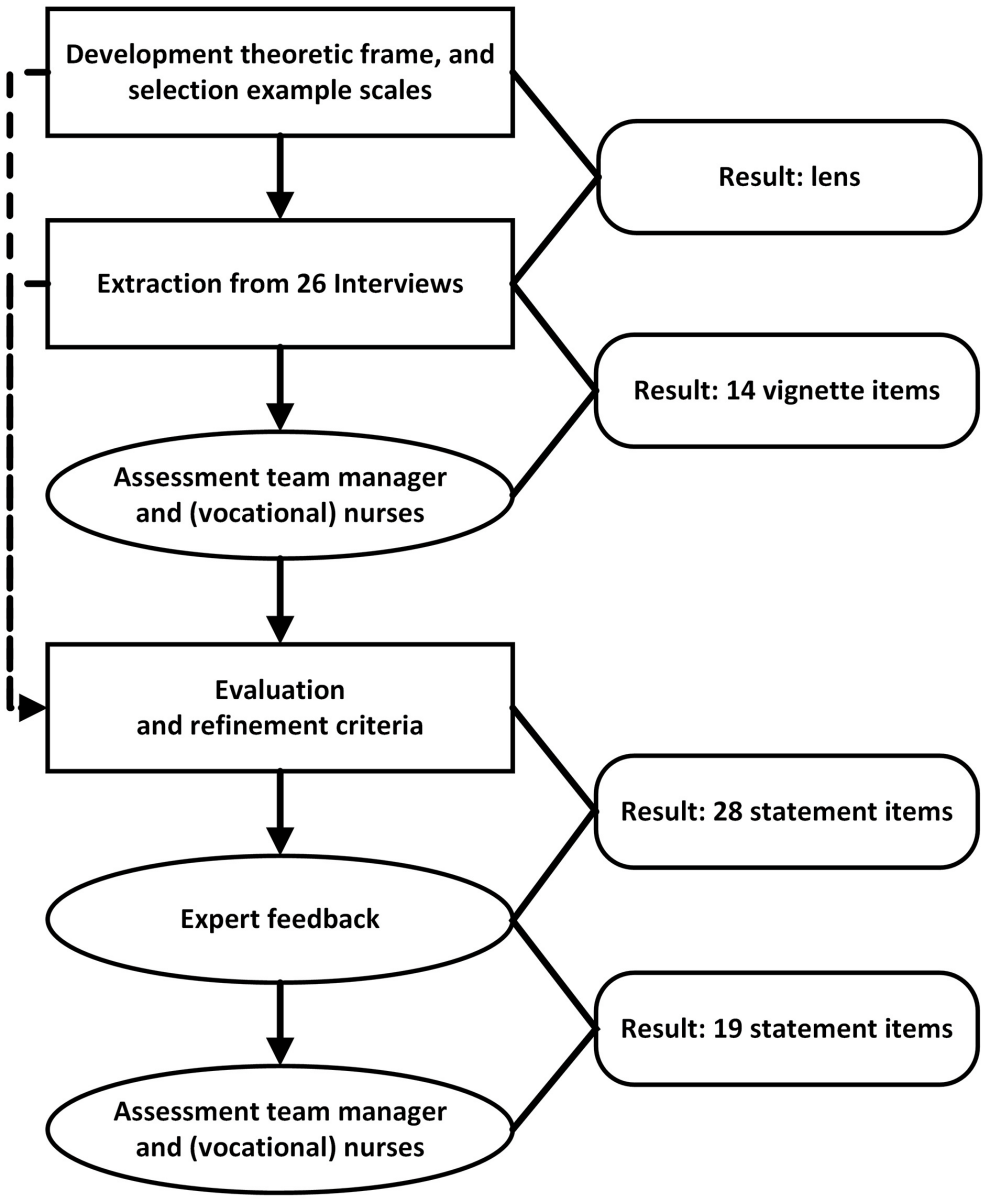
Flowchart development process.

An iterative and co-creative process (see Figure 1) was applied to bridge theoretic principles, expert knowledge, and day-to-day experiences during the development of the Including Personal Boundaries (IPB) scale. From a theoretical perspective, the IPB scale should assess (1) the extent to which nurses feel able to include personal boundaries when anticipating, appraising, and responding to intimate and sexual expressions while (2) minimizing any negative impact on the care relation (in alignment with PCC). Specifically, the inclusion of personal boundaries encourage nurses to step back instead of balancing personal and client needs. How nurses handle client interaction while taking a step back represents the integration of PCC. For instance, are they capable of calmly redirecting a resident's sexual approach, or do they resort to scolding or admonishing. For the daily struggles and successes with including personal boundaries, we relied upon 26 in-depth interviews on the experiences of NH staff (e.g., nurses) with intimate and sexual expressions. The vivid and detailed, yet highly individual, experiences were already coded and analyzed through interpretative phenomenological analysis (IPA) in a previous study (Waterschoot et al., 2021).

### 2.1 Instrument design

Self-efficacy refers to one's confidence in their ability to exert control in a specific domain (Bandura et al., 1999). Through self-reporting, individuals either rate (a) their confidence level in domain-related situations or (b) (dis)agree with statements regarding different domain elements. For the first variant, Bandura (2006) developed a guide for constructing self-efficacy scales with confidence level scales (“can do”). Mackenzie and Peragine (2003) used this guide for their Inventory of Geriatric Nursing, measuring caregivers' confidence to remain calm and resolve problems during general stressful situations in residential dementia care. Statements, the second variant, are used in the General Self-efficacy scale GSE (Schwarzer et al., 1995), and more specifically in the Caring Efficacy Scale CES (Coates, 1997), which measures nurses' ability to express a caring orientation and establish a caring relationship.

In the first development step, we generated 18 vignette items related to intimate and sexual behavior based on the interview experiences (Waterschoot et al., 2021). In line with the Inventory of Geriatric Nursing the items were divided among situational domains and received a similar confidence scale (Mackenzie and Peragine, 2003). The four situational domains were: individual residents, co-residential interactions, family of residents, and colleague's. An example item was: “A spouse tells you in tears that she no longer wants to be intimate with her partner (the client) and asks if you can protect her against his intrusive behavior.” A team manager and two (vocational) nurses evaluated the vignettes. They stressed the relevance of the topics but lacked enthusiasm on multiple items. Their reflections stressed the importance of individual experience and context dependency in coping with this precarious theme. Composing vignettes for every occasion/expression and thinkable context is impossible, so after re-evaluation, we decided upon an item statement design and refined the inclusion and exclusion criteria described below.

Items with statements on the capability to include personal boundaries should consist of (1) a response to intimate or sexual client behavior and (2) a value of self-efficacy. The response is cognitive, emotional, physical, or a combination and occurs during or directly after the interaction. When specified, responses should be relationship and resident centered. Self-efficacy is either phrased as positive or negative. While our initial preference was to encompass the entire spectrum of nurses' experiences with intimate and sexual behavior, we found that the previously defined subdomains incorporated multiple subthemes related to organizational culture, various interpersonal relations, and ethical dilemmas. Examples include instances where people with dementia fail to recognize their spouse or develop romantic feelings for co-residents while being married. To sharpen the focus on the nurse-resident relationship, we excluded both triangular relationships and decision-making on facilitating sexual needs from the scale design. Additionally, we removed gender-specific descriptions of residents, such as references to breasts or sperm, from the items.

Considering these criteria, we created an initial 28-item version of the IPB scale. A 6-point scale (1 completely untrue−6 completely true) was chosen to elicit nuanced differences among nurses. To help participants, three rows of equations (−−−, −−, −, +, ++, +++) were added throughout the item list.

We invited five academic, care-educational, and/or clinical experts to review the 28-item version (see Table 1). The experts individually revised the items by commenting on the wording and relevance of each item separately. Relevance was measured through the content validity index of Lawshe (1975), defining items as: “1 essential,” “2 useful but not essential,” or “3 not necessary.” Seven items scored exclusively “1 essential,” and almost all items received detailed feedback on the wording. Experts emphasized the importance of using concise statements without complex language because vocational nurses may not be accustomed to reading them. This development step resulted in a 19-item scale. We asked the team manager and (vocational) nurses to evaluate this version, and they all rated the items as (very) comprehensible. Lastly, a forward-backward translation from Dutch to English was applied for international purposes.

**Table 1:** Experts.

**Profession**	**Recent experience**		
	**NH practice**	**Scientific research**	**Training and educating**
Sexologist	x		x (p)
Sexologists, PhD	x	x	
Psychologist	x	x	x (p)
Lecturer			x (s) +
Lecturer			x (s) +

(p) is professionals (s) is students.

+Curriculum development on intimacy and sexuality for vocational nurses.

### 2.2 Participants

Five Dutch care organizations that partner in the Academic Collaborative Center Older Adults (Luijkx et al., 2020) participated in the study. These organizations possess multiple nursing homes with psychogeriatric units organized as group homes. Residents in a group home receive dedicated care from care assistants (e.g., welfare supporters, hostesses), licensed vocational nurses (LVN), and registered nurses (RN). The inclusion criteria were: (1) (trainee) vocational and registered nurses, and (2) working in psychogeriatric group homes where at least half of the residents are diagnosed with dementia. In the Netherlands, care assistants supervise the group home living rooms. As they are, for example, not licensed to bathe or (un)dress residents, they are excluded from the study (Welzijn, 2023). Nurses from 25 nursing home locations throughout the Netherlands have been invited to complete an anonymous paper survey on intimacy and sexuality in dementia care, starting with the 19 IPB questions.

Two hundred ninety-seven surveys met both inclusion criteria and were returned with a signed informed consent form (see Table 2 for details). Most participants identified as female (94%) and worked as a vocational nurse (73%). The average age was 43 years (SD 13.64), and the average work experience in care was 17 years (SD 12.28) (see Table 3 for details). Of these surveys, the IPB scale of 88% is complete (*n* = 260), 9% has one or two missing values (*n* = 27), and the remaining 3% has more than two missing values (*n* = 10).

**Table 2:** Response.

**Response**		***n***
Returned surveys		371
Excluded:		
	No informed consent	*17*
	Not a (trainee) nurse	*57*
Included		297
Complete IPB scale		260

**Table 3:** Demographic characteristics for all participants (*n* = 297).

**Age**	**Mean (SD)**
Years	43 (13.64)
**Work experience**	**Mean (SD)**
Years	17 (12.28)
**Gender (identify by)**	***n*** **(%)**
Female	279 (93.9%)
Male	16 (5.4%)
Non-binary	0 (0.0%)
Other, namely	0 (0.0%)
Prefer not to say | no answer	2 (0.7%)
**Role**	***n*** **(%)**
Trainee nurse	31 (10.4%)
Licensed Vocational Nurse (LVN)	218 (73.4%)
Registered Nurse (RN)	48 (16.2%)

### 2.3 Ethical considerations

Prior to the start of the study, ethical approval from the Ethics Review Board (ERB) of Tilburg University (TSB_RP769) was obtained. Ethical committees of the five participating organizations approved the study as well. An information letter informed eligible participants about the nature and voluntary character of the study. Individual consent was received through an informed consent form.

### 2.4 Statistical analyses

Analyses were carried out using SPSS statistics v.27. The scores of the IPB were subjected to analysis to (1) explore the dimensional structure, (2) examine the reliability, and (3) reduce item redundancy (i.e., decrease items). Field's (2018) stepwise procedure guided a Principal Component Analysis (PCA). PCA is performed on respondents who completed all IPB questions as (1) completeness is required to discover item structure, (2) sample amount is still sufficient, and (3) sampling adequacy did not increase through imputation (Field, 2018).

## 3 Results

In general, all included (*n* = 297) participants responded well to the IPB (19 items). There was no indication that specific items were skipped or neglected (see Table 4). Responses for every item covered the full range of response categories except for answer “1 completely untrue” for items 5 and 10. Skewness and Kurtosis are acceptable (-2 to 2), except the scores for I6 and I11^R^ peaked (kurtosis +2). The overall mean of the 13 positive items is 4.89, and of the six negative phrased items before reversing is 2.58.

**Table 4:** Items of the IPB.

**Items**		**Mean *(SD)***	**Floor (*n*, %)**	**Ceiling (*n*, %)**	**Skewness**	**Kurtosis**
I1	I can quickly sense when a client is seeking intimacy or sexuality with me	4.78 (0.97)	1 (3, 1%)	6 (66, 22.2%)	−1.00	1.76
I2^*R*^	I ignore sexual behavior and hope that it will just go away on its own	4.40 (1.40)	1 (13, 4.4%)	6 (71, 23.9%)	−0.77	−0.19
I3	It is easy to set my boundaries when it comes to sexual behavior of clients	4.87 (1.12)	1 (4, 1.3%)	6 (100, 33.7%)	−1.05	0.90
I4	If a client becomes aroused during care, I am able to react respectfully	5.14 (0.92)	1 (1, 0.3%)	6 (122, 41.1%)	−1.10	1.36
I5	It is easy to set my boundaries when it comes to intimate behavior of clients	4.99 (0.95)	2 (1, 0.3%)	6 (108, 36.4%)	−0.60	−0.52
I6	I listen to my gut and choose for myself whether to hug a client	5.26 (0.98)	1 (3, 1.0%)	6 (157, 52.9%)	−1.58	3.08
I7	If I do not want to be touched, I can reverse the client's behavior in a controlled manner	4.83 (0.95)	1 (2, 0.7%)	6 (77, 25.9%)	−0.79	1.15
I8	By watching my posture when bathing a client, I can prevent unwanted touching	4.77 (1.12)	1 (6, 2.0%)	6 (81, 27.3%)	−1.05	1.28
I9^*R*^	I avoid clients who have romantic feelings for me	3.96 (1.42)	1 (23, 7.7%)	6 (41, 13.8%)	−0.47	−0.44
I10	I trust my skills for dealing with inappropriate sexual behavior of clients	5.09 (0.81)	2 (3, 1.0%)	6 (97, 32.7%)	−0.86	1.12
I11^*R*^	I often have the feeling that it's my fault when a client becomes aroused during care	5.34 (0.93)	1 (2, 0.7%)	6 (166, 55.9%)	−1.71	3.63
I12	I am able to provide care again to a client who has previously approached me in a sexual way	4.88 (1.11)	1 (5, 1.7%)	6 (97, 32.7%)	−1.19	1.58
I13	I stay calm when sexual remarks are made because I know how I should react	4.70 (1.06)	1 (3, 1.0%)	6 (73, 24.6%)	−0.77	0.70
I14^*R*^	The only way I can provide care to clients who touch a lot and who I don't like is by ignoring my feelings	4.02 (1.46)	1 (18, 6.1%)	6 (51, 17.2%)	−0.39	−0.74
I15^*R*^	I sense that things get out of hand if I give space for sexuality	4.10 (1.43)	1 (13, 4.4%)	6 (54, 18.2%)	−0.41	−0.70
I16^*R*^	I'm afraid I cannot get it out of my head if I accidentally see clients having sex	4.71 (1.31)	1 (6, 2.0%)	6 (100, 33.7%)	−0.95	0.13
I17	Even if I feel uncomfortable around a client, I am able to create a positive atmosphere when providing care	4.65 (1.00)	1 (4, 1.3%)	6 (55, 18.5%)	−0.90	1.53
I18	I make it work out, regardless of how clingy a client acts	4.68 (1.01)	1 (4, 1.3%)	6 (58, 19.5%)	−0.98	1.69
I1 9	I am able to set aside uncomfortable feelings so that clients and their partners can have sex	4.91 (1.07)	1 (3, 1.0%)	6 (97, 32.7%)	−1.07	1.24

Underlined items are omitted from the instrument in subsequent analyses. R items are reverse-coded. Self-efficacy ranges from 1 (completely untrue) to 6 (completely true). Higher scores are considered better. Numbers are rounded.

For the PCA, initial data checks were carried out. The Kaiser-Meyer-Olkin (KMO) measure has a “meritorious” (Kaiser and Rice, 1974) score of 0.862, verifying sampling adequacy. In addition, all KMO values for individual items are well above the acceptable limit of 0.5. Bartlett's Test of Sphericity is significant (< 0.001), indicating enough shared variance between items. The Pearson Correlation coefficient matrix does not indicate over-correlation between items (i.e., multicollinearity or singularity). However, five of the six reversed items (I02^R^, I09^R^, I14^R^, I15^R^, and I16^R^) require attention during further steps as they lack a significant correlation toward positive phrased (non-reversed) items.

Second, to reduce items and create a parsimonious scale, we commenced with PCA with Varimax rotation (i.e., orthogonal rotation) (Schreiber, 2021). The scree plot opted for two or three components. We performed three subsequent analyses (I, II, III), each with a predetermined number of components (3, 2, 1), depicted in Table 5.

**Table 5:** Factor analysis.

**Items/components**		**Analysis I**			**Analysis II**		**Analysis III**
		**A**	**B**	**C**	**A**	**B**	**A**
I05	It is easy to set my boundaries when it comes to intimate behavior of clients	**0.803**			**0.735**		**0.736**
I03	It is easy to set my boundaries when it comes to sexual behavior of clients	**0.748**			**0.645**		**0.652**
I07	If I do not want to be touched, I can reverse the client's behavior in a controlled manner	**0.730**			**0.684**		**0.676**
I10	I trust my skills for dealing with inappropriate sexual behavior of clients	**0.629**	0.332		**0.707**		**0.695**
I04	If a client becomes aroused during care, I am able to react respectfully	**0.605**	0.307		**0.670**		**0.695**
I13	I stay calm when sexual remarks are made because I know how I should react	**0.598**	0.492		**0.769**		**0.794**
I0 8	By watching my posture when bathing a client, I can prevent unwanted touching	**0.536**			**0.453**		0.391
I0 6	I listen to my gut and choose for myself whether to hug a client	**0.488**			**0.477**		0.475
I11^*R*^	I often have the feeling that it's my fault when a client becomes aroused during care	**0.393**			**0.465**		excl.
I0 1	I can quickly sense when a client is seeking intimacy or sexuality with me	**0.372**			**0.405**		0.421
I18	I make it work out, regardless of how clingy a client acts		**0.828**		**0.650**		**0.654**
I17	Even if I feel uncomfortable around a client, I am able to create a positive atmosphere when providing care		**0.759**		**0.624**		**0.626**
I1 9	I am able to set aside uncomfortable feelings so that clients and their partners can have sex		**0.667**		**0.500**		0.529
I12	I am able to provide care again to a client who has previously approached me in a sexual way.	0.327	**0.576**	0.326	**0.589**	0.373	**0.645**
I09^*R*^	I avoid clients who have romantic feelings for me			**0.696**		**0.695**	excl.
I14^*R*^	The only way I can provide care to clients who touch a lot and who I don't like is by ignoring my feelings			**0.656**		**0.629**	excl.
I02^*R*^	I ignore sexual behavior and hope that it will just go away on its own			**0.620**		**0.619**	excl.
I15^*R*^	I sense that things get out of hand if I give space for sexuality			**0.606**		**0.603**	excl.
I16^*R*^	I'm afraid I cannot get it out of my head if I accidentally see clients having sex			**0.467**		**0.492**	excl.
**Sampling adequacy and reliability**							
	KMO	0.862			0.862		0.885
	Cronbach α	0.830	0.764	0.619	0.865	0.619	0.866

PCA with Varimax rotation. excl. means excluded as input. Analysis I, II: rotated factor loadings, Analysis III component loadings. Loadings < 0.3 are suppressed. Minimal inclusion criteria Analysis III is loading of ≥0.6. R items are reverse-coded. Underlined items are omitted from the final instrument, which is depicted in the Appendix. Bold items are included in the component.

In analysis I, component C consists of five reversed items and has an insufficient Cronbach *a* (0.619) (Tavakol and Dennick, 2011). In addition, I04, I10, I12, and I13 cross load (>0.3). In analysis II, one component (A) consists of all positive items plus item I11^R^. The second component (B) consists of the remaining reversed items. Identical to analysis I, the Cronbach *a* of the second component is insufficient. Also, the component score of I11^R^ is on the lower end (0.465), and I12 still cross-loads. Therefore, we excluded all reversed items for analysis III and tested a one-component (i.e., unidimensional) scale. Bolstering a parsimonious scale, we decided upon a high factor loading criterium of 0.6 (Carpenter, 2018). Four items (I01, I06, I08, I19) were excluded by applying this criterion, strengthening the focus on the actual response contrary to items related to anticipation skills (I01, I08) and indirect situations (I19).

Third, the internal reliability of the (remaining) 9-item IPB scale was assessed by considering (a) Cronbach *a* coefficient, (b) corrected item-total correlation, (c) the alpha estimate when an item is dropped from the scale, and (d) inter-item correlations (Table 6). An overall Cronbach *a* of 0.866 indicates “very good” reliability (Hair et al., 2019) while remaining below the threshold (>0.9) of item redundancies (Tavakol and Dennick, 2011). All items contribute positively to overall reliability. The corrected item-total correlations are above the minimum of 0.5 (Hair et al., 2019) and comparable (range 0.532–0.731) (Field, 2018). Inter-item correlations differ, which can indicate underlying subthemes. The inter-item correlation mean is 0.422, which suits the suggestion of Clark and Watson (2016) for a mean between 0.15 and 0.5, where a higher score serves narrower psychological constructs.

**Table 6:** Pearson inter-item correlation matrix and item reliability characteristics.

**Items**	**I3**	**I4**	**I5**	**I7**	**I10**	**I12**	**I13**	**I17**	**Corrected item-total correlation**	**Chronbach *a* if item deleted**
I3	–								0.580	0.854
I4	0.416^**^	–							0.591	0.853
I5	0.697^**^	0.524^**^	–						0.660	0.847
I7	0.465^**^	0.427^**^	0.486^**^	–					0.573	0.854
I10	0.417^**^	0.444^**^	0.489^**^	0.437^**^	–				0.604	0.853
I12	0.332^**^	0.387^**^	0.315^**^	0.334^**^	0.375^**^	–			0.561	0.856
I13	0.481^**^	0.494^**^	0.566^**^	0.485^**^	0.490^**^	0.553^**^	–		0.731	0.839
I17	0.241^**^	0.309^**^	0.300^**^	0.319^**^	0.357^**^	0.399^**^	0.431^**^	–	0.532	0.858
I18	0.243^**^	0.335^**^	0.296^**^	0.291^**^	0.392^**^	0.470^**^	0.523^**^	0.661^**^	0.570	0.855

^**^Correlation is significant at the 0.01 level (two-tailed).

Finally, the IPB 9-item sum score was calculated for the included participants (*n* = 297). To address missing data, we examined missing values at respondent level [total missing(s) 0: 94.6%, 1: 2.7%, 1+: 2.7%], and at item level (min. 0.3% max. 2%). Little's MCAR Test confirmed that all 9 items were missing completely at random. Subsequently, we chose to calculate the IPB 9-item sum score for respondents with maximum of one missing value (missing replaced through “sum by mean” in SPSS). The average sum score for the 9-item list, based on data from 289 participants, was 43.84 (SD 6.16, range 9–54), indicating that, in general, nurses appear to feel confident. Confidence tends to be higher for nurses identifying as male or those with at least 5 years of work experience (see Table 7 for details).

**Table 7:** Sum scores IPB-9 items mean (SD).

	**Type of nurse**			**Years of work experience** ^*^		**Gender** ^**^ **(binary)**	
	**Trainee** ***(n** = **30)***	**Licensed vocational** ***(n** = **211)***	**Registered** ***(n** = **48)***	<**5 years** ***(n** = **72)***	**5**+ **years** ***(n** = **217)***	**Female** ***(n** = **272)***	**Male** ***(n** = **15)***
IPB 9-items	41.40 (7.90)	44.01 (5.98)	44.60 (5.41)	42.41 (6.91)	44.32 (5.82)	43.61 (6.17)	47.83 (4.85)

^*^p < 0.05. ^**^p < 0.001.

## 4 Discussion

This paper presents the development of a new scale, the Including Personal Boundaries (IPB) scale (see “Appendix” section). The overall score of the summed-up items reveals nurses' confidence in their capabilities to include personal boundaries when confronted with the intimate and sexual behavior of residents with dementia. An iterative and co-creative process integrated theoretic principles, day-to-day experiences, and clinical expertise to develop the scale. This process was particularly important as attention toward personal boundaries is scarce in the (PCC) literature, even though it is a crucial aspect of nurses' daily work, and intimate and sexual behavior substantially influences the care relation (Mcguire et al., 2016).

The composed 9-item IPB scale exhibits robust psychometric properties. Internal reliability measures, for example, showed very good results. Through a principal component analysis, nine positively oriented items were selected. As a result, the scale conveys a sense of empowerment resembling the positively oriented GSE scale. The distribution of items related to sexual acts vs. intimate/unspecified client behavior remained well-balanced. Therefore, the questionnaire is broad in its interpretation of intimate and sexual behavior, which is important because care professionals evaluated the vignette items as too narrow and individually phrased. Due to the broadness, the IPB scale might also be relevant in other residential care environments for people with cognitive impairments who require daily care, such as people with profound intellectual disabilities. As to our knowledge, no similar scales are available.

Other studies have shown that general self-efficacy increases nurses' feelings of empowerment (Keyko et al., 2016) and moderates, together with personality type, the effect of stress on nurses' job-related burnout (Yao et al., 2018). We believe the IPB scale can be used for (self-) assessment in clinical practice and for trainee nurses. Although sexual expression is a very private activity, and nurses are preferably not present when expressed, NH residents with severe dementia can show intimate and sexual behavior in their presence for various reasons. Nurses' capability to include personal boundaries in these situations benefits their wellbeing and the care relation (Waterschoot et al., 2021). Viewpoints in current research on intimacy and sexuality in dementia care are relatively one-dimensional as, often, nurses are either portrayed as victims of sexual harassment or accountable for unmet resident needs due to inadequate attitudes or lack of knowledge (Haesler et al., 2016; Kontos et al., 2016; Villar et al., 2020; Peisah et al., 2021). Quantitative studies aiming to nuance, bridge differences, or empower nurses might benefit from applying the IPB scale.

This study has limitations. First, the item development process was in Dutch, and translation was completed after development. While it cannot be ruled out, we do not expect limitations for international use because Dutch culturally sensitive practices, such as partners having sex in the NH or the possibility of specialized sex workers for residents with specific needs, were purposefully excluded from the IPB scale. Testing, however, is necessary to confirm this assumption. Second, although the IPB scale covers a broad range of experiences, it does not cover the full range of (more extreme) incidents related to intimacy and sexuality, which often relate to other themes such as aggression. This was a deliberate decision based on professional feedback; however, these incidents are probably more challenging for nurses in practice. Third, the pen-and-paper survey, although convenient for data collection, was non-randomized. Where feasible, we recommend employing randomized methodologies in future studies to enhance reliability. Finally, as this study presents the development phase of the PB scale, participants initially completed a 19-item version, later reduced to a 9-item version after statistical analysis. Consequently, more research is needed, particularly regarding the validity of the final IPB scale. For instance, while internal consistency is a necessary criterion, it alone does not guarantee unidimensionality (Clark and Watson, 2016), therefore additional verification of potential subscales is necessary. Unfortunately, criterion validity assessment was not possible as we did not include a golden standard instrument in the survey, such as the general self-efficacy scale. To our knowledge no comparable scale exists to measure the same construct. It is, however, most interesting to discover how nurses IPB scores correlate with their psychosocial work experiences and perceived relationship quality with residents with dementia. Overall, we anticipate that additional research will establish a solid foundation for interpreting IPB scores and eventually the development of normative scores.

## 5 Conclusion

An instrument to assess nurses' confidence levels in their capabilities to include personal boundaries was missing in the context of intimate and sexual behavior and in relation to PCC. The IPB scale is a quick and simple tool that could facilitate the assessment of self-efficacy as part of nurses' competence to balance personal and residential needs. The results of the pilot are promising. Further work on testing and validating the IPB scale is highly recommended.

## Data availability statement

The data presented in this article are not readily available due to confidentiality and in accordance with the ethical approval for the studies. Requests to access the dataset should be directed to k.waterschoot@tilburguniversity.edu.

## Ethics statement

The studies involving humans were approved by Ethics Review Board (ERB) of Tilburg University. The studies were conducted in accordance with the local legislation and institutional requirements. The participants provided their written informed consent to participate in this study.

## Author contributions

KW: Writing – original draft. TR: Supervision, Writing – review & editing. AD: Supervision, Writing – review & editing. KL: Supervision, Writing – review & editing.

## Appendix

                     Including Personal Boundaries (IPB) scale

**English**

Dealing with intimate and sexual behavior in clients with dementia *For each statement, choose the answer that best fits your situation*.

1. It is easy to set my boundaries when it comes to sexual behavior of clients.
2. If a client becomes aroused during care, I am able to react respectfully.
3. It is easy to set my boundaries when it comes to intimate behavior of clients.
4. If I do not want to be touched, I can reverse the client's behavior in a controlled manner.
5. I trust my skills for dealing with inappropriate sexual behavior of clients.
6. I am able to provide care again to a client who has previously approached me in a sexual way.
7. I stay calm when sexual remarks are made because I know how I should react.
8. Even if I feel uncomfortable around a client, I am able to create a positive atmosphere when providing care.
9. I make it work out, regardless of how clingy a client acts.

**Dutch**

Omgaan met intiem en seksueel gedrag bij cliënten met dementie *Kies voor iedere stelling het antwoord dat het beste bij jou past*.

1. Het is gemakkelijk om mijn grenzen te bewaken als het gaat om seksueel gedrag van cliënten.
2. Als een cliënt opgewonden wordt tijdens de zorg kan ik hier respectvol op reageren.
3. Het is gemakkelijk om mijn grenzen te bewaken als het gaat om intiem gedrag van cliënten.
4. Als ik niet aangeraakt wil worden kan ik het gedrag van de cliënt gecontroleerd ombuigen.
5. Ik vertrouw op mijn vaardigheden om met ongepast seksueel gedrag van cliënten om te gaan.
6. Aan een cliënt die mij seksueel heeft benaderd, kan ik opnieuw zorg verlenen.
7. Ik voel mij kalm bij seksuele opmerkingen omdat ik weet hoe ik moet reageren.
8. Zelfs als ik mij ongemakkelijk voel bij een cliënt ben ik in staat om een positieve sfeer te creëren tijdens de zorg.
9. Hoe aanhankelijk een cliënt zich ook opstelt, ik kom er wel uit.

*The participants indicate their level of agreement with the statements using a six-item scale, with on the left side “completely untrue,” and on the right “completely true” (in Dutch “volledig onjuist” and “volledig juist”). The minimum score possible for each question is 1, and the maximum is 6. A total score is calculated by adding up each item score (ranging from 9 to 54). The higher the score, the greater one's confidence in their capabilities to include personal boundaries*.
